# Peptide-RNA Coacervates as a Cradle for the Evolution of Folded Domains

**DOI:** 10.1021/jacs.2c03819

**Published:** 2022-07-29

**Authors:** Manas Seal, Orit Weil-Ktorza, Dragana Despotović, Dan S. Tawfik, Yaakov Levy, Norman Metanis, Liam M. Longo, Daniella Goldfarb

**Affiliations:** † Department of Chemical and Biological Physics, Weizmann Institute of Science, Rehovot 7610001, Israel; ‡ Institute of Chemistry, The Hebrew University of Jerusalem, Jerusalem 9190401, Israel; § Department of Biomolecular Science, Weizmann Institute of Science, Rehovot 7610001, Israel; ∥ Department of Chemical and Structural Biology, Weizmann Institute of Science, Rehovot 7610001, Israel; ⊥ Casali Center for Applied Chemistry, The Hebrew University of Jerusalem, Jerusalem 9190401, Israel; # The Center for Nanoscience and Nanotechnology, The Hebrew University of Jerusalem, Jerusalem 9190401, Israel; ∇ Earth-Life Science Institute, Tokyo Institute of Technology, Tokyo 152-8550, Japan; ○ Blue Marble Space Institute of Science, Seattle, Washington 98104, United States

## Abstract

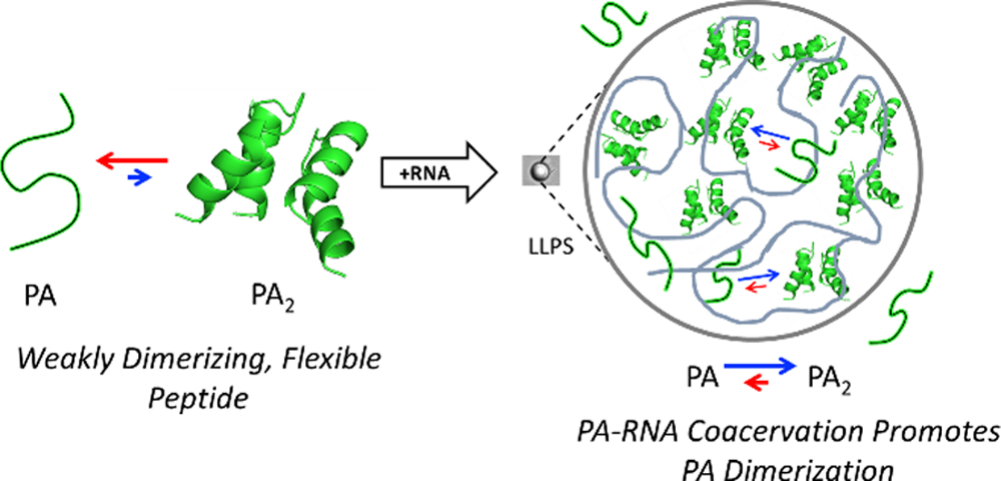

Peptide-RNA coacervates can result in the concentration and compartmentalization of simple biopolymers. Given their primordial relevance, peptide-RNA coacervates may have also been a key site of early protein evolution. However, the extent to which such coacervates might promote or suppress the exploration of novel peptide conformations is fundamentally unknown. To this end, we used electron paramagnetic resonance spectroscopy (EPR) to characterize the structure and dynamics of an ancient and ubiquitous nucleic acid binding element, the helix-hairpin-helix (HhH) motif, alone and in the presence of RNA, with which it forms coacervates. Double electron–electron resonance (DEER) spectroscopy applied to singly labeled peptides containing one HhH motif revealed the presence of dimers, even in the absence of RNA. Moreover, dimer formation is promoted upon RNA binding and was detectable within peptide-RNA coacervates. DEER measurements of spin-diluted, doubly labeled peptides in solution indicated transient α-helical character. The distance distributions between spin labels in the dimer and the signatures of α-helical folding are consistent with the symmetric (HhH)_2_-Fold, which is generated upon duplication and fusion of a single HhH motif and traditionally associated with dsDNA binding. These results support the hypothesis that coacervates are a unique testing ground for peptide oligomerization and that phase-separating peptides could have been a resource for the construction of complex protein structures *via* common evolutionary processes, such as duplication and fusion.

## Introduction

Liquid–liquid phase separation (LLPS) is an important biological process^1−3^ with notable relevance to the primordial world,^4,5^ particularly as a way for simple peptides and nucleic acids to achieve compartmentalization and concentration. Little is known, however, about peptide-RNA coacervates as a site for the evolution of protein structure and function, and it is uncertain to what extent phase-separating polypeptides were a resource for the evolution of more complex protein structures.^6^ Even the effect of the coacervate context on protein structure and oligomerization is unclear: On the one hand, coacervates are characterized by a network of weak, transient, multivalent interactions between polypeptide and RNA molecules^1,7^ that, collectively, may favor extended peptide conformations—especially for peptides with marginal folding energies. After all, unstructured peptides can readily form coacervates with nucleic acids^8^ (though they do not necessarily do so). On the other hand, the high concentrations and reduced water activity within a coacervate, as well as interactions with the RNA, may promote peptide oligomerization and structural collapse.

Probing the relationship between defined oligomeric states, folding, and phase separation is of fundamental importance to our understanding of protein evolution. Oligomeric states are now recognized as a defining feature in the early evolution of symmetric and repetitive protein architectures,^9^ including the β-trefoil,^10^ the β-propeller,^11,12^ the double-ψ β-barrel,^13^ and the P-Loop NTPases.^14^ It is through oligomerization that short peptides can achieve structures of *sufficient complexity and stability* to execute biological functions. Consequently, the extent to which peptide-RNA coacervates permit the formation of stable, well-defined oligomeric states has a significant impact on the role of coacervates in the evolution of structured domains.

To address these questions, we consider the early evolution of the helix-hairpin-helix (HhH) motif—an ancient and ubiquitous nucleic acid binding element that, upon duplication and fusion, adopts a rotationally symmetric architecture called the (HhH)_2_-Fold.^15−17^ It has been previously observed that simple HhH motif peptides phase-separate in the presence of polyU and that duplication of this peptide results in the formation of an (HhH)_2_-Fold that binds to double-stranded DNA (dsDNA).^17^ However, whether a single, simple HhH motif can dimerize to recapitulate the (HhH)_2_-Fold is unclear and, even more mysterious, is the structure and oligomerization state of the HhH motif within peptide-RNA droplets. Using a combination of electron paramagnetic resonance (EPR) techniques applied to site-specifically labeled peptides,^18,19^ we can now shed light on these processes. Continuous wave (CW) EPR, which reports on the motional freedom of the peptide, allowed free and RNA-bound peptides to be distinguished; pulse EPR and echo-decay measurements reported on local peptide concentrations; and double electron–electron resonance (DEER) spectroscopy probed peptide conformations and dimerization.

We observe that an HhH motif-containing peptide is able to dimerize into structures that are consistent with the (HhH)_2_-Fold, in agreement with both an AlphaFold2 model of the HhH motif dimer and current models of symmetric protein evolution, in which an oligomeric state precedes a single-chain domain.^10−12^ Dimerization, however, was not restricted to just aqueous buffer: the peptide within peptide-RNA coacervates was also observed to adopt a dimeric structure. Moreover, dimerization was promoted by association with RNA. We argue that coacervates may have been an ideal testing ground for the emergence of stable oligomers, conformational states that could later be selected for and stabilized by duplication and fusion events.^20^ In this way, droplet-associated peptides could have been a key starting point for the evolution of folded protein domains.

## Experimental Section

### Peptide Synthesis

#### Materials

Buffers were prepared using Milli-Q water (Millipore, Merck). Na_2_HPO_4_·12H_2_O, ethanedithiol (EDT), triisopropylsilane (TIPS), and 4-maleimido-TEMPO were purchased from Sigma-Aldrich (Rehovot, Israel). (Tris(2-carboxyethyl)phosphine (TCEP) was purchased from Merck. All Fmoc-amino acids were obtained from CS Bio Co. (Menlo Park, CA), Matrix Innovation (Quebec City, Canada), or Chem-Impex Inc. (Bensenville, IL, United States), with the following side chain-protecting groups: Arg(Pbf), Glu(OtBu), Gly(OtBu), Ser(tBu), Thr(tBu), Tyr(tBu), (Pbf = 2,2,4,6,7-pentamethyl-2,3-dihydrobenzofuran-5-sulfonyl). TentaGel R RAM resin (loading 0.19 mmol/g) was purchased from Rapp Polymer GmbH (Germany). 1-[Bis(dimethylamino)methylen]-5-chlorobenzotriazolium 3-oxide hexafluorophosphate, *N,N,N′,N′*-tetramethyl-O-(6-chloro-1H-benzotriazol-1-yl)uronium hexafluorophosphate (HCTU) was purchased from Luxembourg Biotechnologies Ltd. (Rehovot, Israel). All solvents, *N,N*-dimethylformamide (DMF), dichloromethane (DCM), acetonitrile (ACN), *N*,*N*-diisopropylethyl amine (DIEA), trifluoroacetic acid (TFA), piperidine (Pip), dimethylsulfoxide (DMSO), were purchased from Bio-Lab (Jerusalem, Israel) and were peptide synthesis, HPLC, or ULC-grade.

#### Synthesis of Peptides Containing Cysteine for EPR Studies

Peptides were prepared by an automatic peptide synthesizer (CS136XT, CS Bio Inc. CA) typically on 0.25 mmol Rink amide resin (RAPP Polymer, loading 0.19) scales. Fmoc-protected amino acids (2 mmol in 5 mL DMF) were activated with HCTU (2 mmol in 5 mL DMF) and DIEA (4 mmol in 5 mL DMF) for 5 min and allowed to couple for 25 min, with constant shaking. Cys residues were inserted at different positions to allow for reaction with the labeling reagent 4-maleimido-TEMPO (M-TEMPO). Arg residues were doubly coupled. Fmoc-deprotection was carried out with 20% Pip in DMF (2 × 5 min).

#### Cleavage and Deprotection

The peptide resins were washed with DMF and DCM and dried under vacuum. The dried peptide resins were deprotected and simultaneously cleaved using a TFA/water/thioanisole/TIPS/EDT (92.5:1.5:1.5:1.5:1.5) cocktail for 4 h. The cleavage mixtures were filtered, and TFA was evaporated with N_2_-bubbling to a minimum volume, to which an eightfold volume of cold ether was added dropwise. The precipitated crude peptides were centrifuged (5000 rpm, 10 min), ether was removed, and the crude peptide was dissolved in ACN/water (1:1) containing 0.1% TFA and was further diluted to ca. 25% ACN with water and lyophilized. The yields of the crude peptides were PA-12: 411 mg, PA-29: 1010 mg, SA-12: 265 mg, and PA-2/12: 600 mg.

#### Purification

50 mg of each peptide was dissolved with 25% ACN and 75% water and then purified by preparative reversed-phase HPLC (RP-HPLC) using an XSelect C4 column using a gradient of 30–50% B over 42 min. The yields were as follows: PA-12: 16 mg (32% yield); PA-29: 13 mg (26% yield); SA-12: 13 mg (26% yield); and PA-2/12: 7.5 mg (15% yield). The HPLC analyses were carried out on a C4 analytical column, and the collected fractions were characterized by mass spectrometry (Figures S1–S4).

#### Labeling of the Peptides and Purification

1 mM of peptide was dissolved in 0.1 M phosphate buffer, pH ∼ 7. Then 10 equiv of TCEP was added to reduce any disulfide in the peptides. A stock solution of 10 equiv of 4-maleimido-TEMPO dissolved in DMSO was also prepared. While stirring the peptide solution, 5 equiv of the label was added dropwise. The reaction was carried out in an oxygen-free environment at room temperature and completed after approximately 5 h (Thiol-Reactive Probe Labeling Protocol provided by Thermo Fisher Scientific). Labeled peptides were purified by an RP-HPLC XSelect C4 column using a gradient of 30–50% B over 42 min. Finally, the purified, labeled peptides were analyzed using HPLC on a C4 analytical column and characterized by mass spectrometry (Figures S1–S4) to confirm their purity and identity. Yields were about 70–85% after the purification step.

#### High Performance Liquid Chromatography

Analytical RP-HPLC was performed on a Waters Alliance HPLC with 220 and 280 nm UV detection using an XBridge BEH300 C4 column (3.5 μm, 130 Å, 4.6 × 150 mm). Preparative RP-HPLC was performed on an XSelect C4 column (5 μm, 130 Å, 19× 250 mm). The flow rates were 1 mL/min (analytical) and 10 mL/min (preparative). Linear gradients of ACN (with 0.1% TFA, eluent B) in water (with 0.1% TFA, eluent A) were used for all systems to elute-bound peptides.

#### Electrospray Ionization Mass Spectrometry

Electrospray ionization mass spectrometry was performed on an LCQ Fleet Ion Trap mass spectrometer (Thermo Scientific). Peptide masses were calculated from the experimental mass to charge (*m*/*z*) ratios from the observed multiply charged species of a peptide. Deconvolution of the experimental MS data was performed with the software package MagTran v1.03.

### Microscopy

Optical microscopy images of phase-separated samples were taken at the de Picciotto Cancer Cell Observatory Life Sciences Core Facilities (Weizmann Institute of Science) using a 100× objective (oil immersion) on a Leica DMI8 microscope with differential interface contrast. We used 1–2 μL of the sample to make a small drop in an imaging chamber produced by attaching a coverslip to a clean glass slide using a thin strip of double-sided tape (AJ Sign World). Images were processed using the software package Fiji (NIH).

### Sample Preparation for EPR

Lyophilized, spin-labeled peptides were dissolved in Milli-Q water and centrifuged at 4000 rpm for 10 min to remove insoluble aggregates. The soluble fractions were used to prepare stock solutions of 250–500 μM peptide. Labeled peptide concentrations were determined by comparing the area under the curve of the EPR signal against that of standard solutions of 10–285 μM TEMPO. For the EPR measurements in D_2_O, 200 μL of the stock solution was lyophilized and re-dissolved in D_2_O. The concentration of the unlabeled peptide stock solutions was determined using the bicinchoninic acid assay (Thermo Fisher Scientific) and ranged from 0.8 to 1.2 mM peptide. A stock solution of 500 mM MES, pH 5.6 was used to adjust all samples to a final concentration of 50 mM MES, pH 5.6. Aliquots of 10 mg/mL polyuridylic acid (polyU; Sigma Aldrich) were prepared in Milli-Q water and diluted as necessary. For CW-EPR measurements, a quartz capillary of 0.6 mm i.d. and 0.84 mm o.d. was filled with 7–8 μL of the sample and sealed with Critoseal (Fisherbrand, Fisher Scientific) at one end. A single capillary was placed in an X-band EPR tube of 2 mm i.d. and 2.4 mm o.d. for measurements, except for spin concentration below 50 μM, where two capillaries were used.

Each sample was freshly prepared and the time to collect CW-PR specte CW-EPR data was 30–40 min. For samples forming droplets, we could observe turbidity, but within the above time, we did not observe separation into a condensed and a dilute phase. Likewise, optical microscopy samples prepared on slides, did not form clear phase boundary within 30–45 min.

For pulse EPR measurements, 5–6 μL of peptide in D_2_O with 20% glycerol-*d*_8_ was introduced into a quartz capillary of 0.9 mm i.d. and 1.1 mm o.d. and sealed with Critoseal at one end. The samples with polyU (and thus, the potential for coacervates) were flash-frozen in liquid nitrogen after incubating at room temperature for 15–20 min. Samples without polyU were directly inserted into the spectrometer at 25 K.

### CW-EPR Spectroscopy

CW-EPR spectra were recorded on an Elexsys E500 X-Band (9.5 GHz) Bruker spectrometer using a high sensitivity resonator at room temperature. The following parameters were used: 0.1 mT field modulation amplitude, a 15 mT scan range, and 20 mW microwave power. Each scan was 42 s and at least 9 scans were accumulated for each data set. For samples with a high concentration of polyU or a low concentration of labeled peptide, at least 25–36 scans were collected to improve the signal to noise (*S*/*N*) ratio. Simulations of the spectra were performed using the “Chili” routine of EasySpin (www.easyspin.org).^21^ The parameters for simulations are given in Tables S1 and S2.

### Pulse EPR Spectroscopy

Pulse EPR measurements were carried out at the W band (94.9 GHz) on a home-built spectrometer at 25 K.^22−24^ Echo intensities were measured with a Hahn echo sequence (π/2_νobs_ – τ – π_νobs_ – τ – echo) using π/2 and π pulses of 20–25 ns and 40–50 ns, respectively, and τ = 500 ns. Echo decays were measured under the same conditions with an initial τ value of 500 ns. DEER measurements were recorded using the four-pulse DEER sequence (π/2_νobs_ – τ_1_ – π_νobs_ – (τ_1_ + *t*) – π_νpump_ – (τ_2_-*t*) – π_νobs_ – τ_2_ – echo)^25,26^ with a chirp pump pulse^23^ using eight-step phase cycling and monitoring the echo intensity as a function of increasing *t*. The general setup is given in Figure S5. The maximum intensity of the nitroxide spectrum was set to 94.9 GHz, the observer pulses were set to 94.85 GHz, and the π/2 and π pulse durations were 35–40 and 70–80 ns. The chirp pump pulse frequency was 94.88–94.98 GHz with a duration of 128 ns. The repetition time was 10 ms. The samples without polyU and a concentration above 50 μM were averaged for 1–5 h. All the samples with a concentration ≤50 μM and all the samples in the presence of polyU were averaged for 6–14 h. The DEER data were analyzed using the DeerAnalysis 2018^27^ program with Tikhonov regularization^28^ and also verified with DEERNet.^29^ For DeerAnalysis, the background was fitted with a homogenous three-dimensional distribution. The calculated distance distributions were obtained from mtsslSuite^30^ using maleimide TEMPO (M-TEMPO) as the ligand, and 200 rotamer conformations were generated using clash treatment set to “loose”.

### Molecular Dynamics Simulations

We performed atomistic molecular dynamics (MD) simulations of the (HhH)_2_-Fold dimer using GROMACS (v. 2020.6).^31^ The force field parameters for the protein, SPC water, and ions were derived from the AMBER99SB-ILDN force field. For those models including RNA, the AMBER99-BSC force field^32^ was used. All structures were placed in a dodecahedral box and solvated. Sodium and chloride ions were added to a concentration of 0.125 M, with slight adjustments to neutralize the overall charge. All structures were subject to minimization and NVT and NPT equilibration. The initial atomic coordinates were taken from the predicted AlphaFold2 model for the PA dimer, calculated with ColabFold using MMseqs2.^33^ The SA dimer was modeled by introducing mutations into the PA dimeric structures. Initial RNA structures were ideal single-stranded B-RNA, generated by Coot.^34^ For each system, three 500 ns simulations were performed and used to estimate RMSDs and α-helical propensities.

## Results

### Precursor-Arg (PA): A Primordial HhH Peptide

The basis for the work presented here is the construct Precursor-Arg (PA).^17^ PA consists of a single helix-hairpin-helix (HhH) motif and is constructed from an alphabet of only 10 amino acid types. The sequence of PA (Table 1) is biased specifically for those amino acids that were thought to be present on the early earth^35^ and is the result of an ancestor sequence reconstruction of the (HhH)_2_-Fold protein family followed by an experimental deconstruction.^17^ PA has two positively charged patches—located at the N-terminus of the first α-helix (residues 1–4, RIRR) and on the second α-helix (residues 19–23, RLARR)—and forms coacervates with polyU.^17^ At the pH 5.6 condition used in this study, PA has an estimated charge of +3.2 (with the labeled variant of PA, PA-12, it has an estimated charge of +4.2).

**Table 1:**
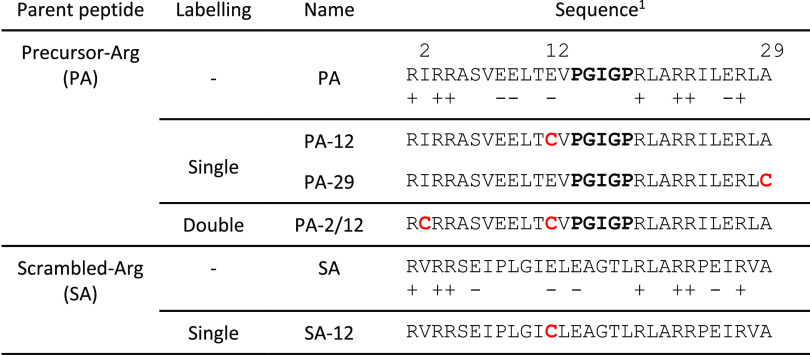
Peptide sequences

a The PGIGP nucleic acid-binding loop is shown in bold. Cys residues spin-labeled with M-TEMPO are shown in red.

**Figure 1:**
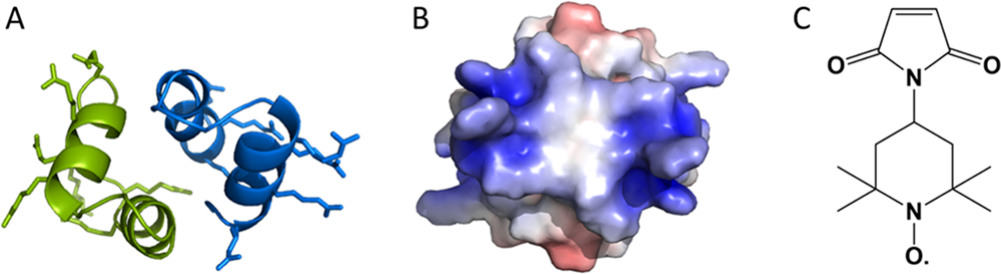
(A) AlphaFold2 model of the PA dimer adopts the (HhH)_2_-Fold. The protein backbone is shown in the cartoon representation, and the seven Arg side chains of each PA molecule are shown as sticks. (B) Surface electrostatic potential of the PA dimer, calculated with the APBS Electrostatics function in PyMOL (pymol.org). (C) Structure of the nitroxide spin label, M-TEMPO, used in this study. Attachment to the protein occurs when a Cys thiol reacts with the maleimide double bond to form a thioester linkage. All structure figures were rendered in PyMOL.

Using the ColabFold implementation of AlphaFold2,^33^ a model of a putative PA dimer was predicted (Figure 1A). As expected, the resulting structure is consistent with the (HhH)_2_-Fold and has a two-fold axis of rotational symmetry. The electrostatic potential surface of the AlphaFold2 model reveals the presence of two distinct patches of positive charge density, as well as two regions of negative charge density (Figure 1B). The dimerization potential of PA was further confirmed by MD simulations (Figure S6). The dimeric structure of PA persisted and maintained its α-helical content, whereas the dimer formed by a control peptide with a scrambled sequence (Scrambled-Arg or SA; Table 1) showed significantly lower conformational stability, including α-helix unwinding and a smaller interface due to partial dissociation. Previously, it has been shown that SA, which preserves the locations of the Arg residues (but otherwise has a scrambled sequence, including the Glu residues, with the same amino acid composition as PA) precipitates with polyU rather than forms coacervates.^17^ A titration with trifluoroethanol, which induces α-helical structure in peptides, followed by circular dichroism spectroscopy revealed that SA has a lower α-helical propensity than PA.^17^

**Figure 2:**
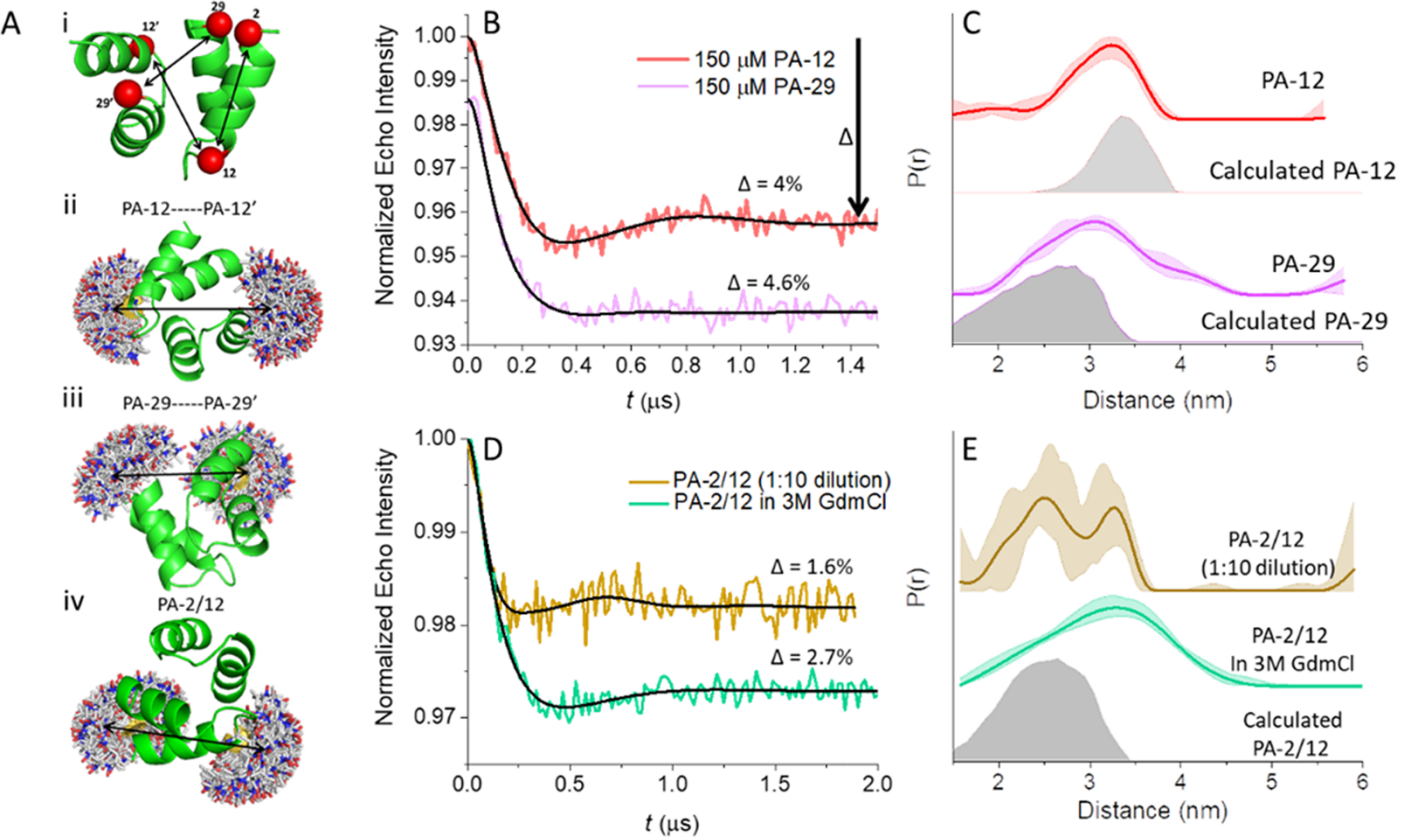
(A) AlphaFold2 model of the PA dimer with labeled positions annotated with a red sphere (i). The arrows indicate the dimer distances measured with PA-12–PA-12′ (ii) and PA-29–PA-29′ (iii) and a α-helical distance measured with PA-2/12 (iv). The clouds of conformations in ii–iv show the predicted rotamers of the nitroxide spin label calculated from mtsslSuite for positions 12 and 12′ (ii), 29 and 29′ (iii), and 2 and 12 (iv). The arrows represent one of the distances contributing to the overall distance distribution. (B) Background-corrected DEER traces of 150 μM PA-12 (red) and PA-29 (magenta) and the corresponding fit (black). The arrow in B indicates the modulation depth (Δ) for PA-12. (C) Distance distribution from fits of the DEER traces (Panel B) and the calculated distribution from mtsslSuite (gray). (D) Background-corrected DEER trace of spin-diluted PA-2/12 (dark yellow; 20 μM labeled PA-2/12, 200 μM total PA concentration) and PA-2/12 (100 μM) in 3 M guanidinium chloride (GdmCl; green). (E) Distance distributions from fits of the DEER traces in Panel D and the calculated distribution from mtsslSuite (gray). The shading around the bold lines of the distance distributions in Panels C and E represents the upper and lower error estimates of the distribution (mean value ±2 standard deviations) calculated from all trials of the background correction parameters by Tikhonov regularization in DeerAnalysis. The primary DEER traces for Panels B and D are shown in Figure S8 and Figure S13C, respectively**.**

Using the AlphaFold2 dimer model as a guide, we selected cysteine mutation sites for spin labeling with M-TEMPO (Figure 1C). The primary considerations for site selection were to avoid mutations in the hydrophobic core and to preserve the positively charged amino acids, which are likely important for interaction with RNA and phase separation. The sequences of the resulting constructs are listed in Table 1, the AlphaFold2 dimer model along with the cloud of rotamers of grafted M-TEMPO spin labels are shown in Figure 2A, and the calculated distance distributions obtained with mtsslSuite^30^ are shown in Figure 2C**.** For control experiments, equivalent sites of the SA peptide were mutated and labeled. The naming convention for spin-labeled peptides is the parent peptide followed by the site (or sites) of spin labeling. For example, PA-12 refers to the Precursor-Arg peptide in which position 12 has been modified with a cysteine and attached to an M-TEMPO spin label.

### PA Forms Dimers in Solution

To determine if dimers, like those predicted by AlphaFold2, can be detected in aqueous buffer, PA-12 and PA-29 (Table 1) were subjected to a detailed EPR analysis. First, CW-EPR spectra of spin-labeled peptides were collected at room temperature and the resulting spectra (Figure S7) were indicative of a nitroxide moiety undergoing fast motion, as is typical for spin-labeled peptides, and distinct from free spin label in solution. The spectrum for PA-29 indicated faster dynamics than that of PA-12, consistent with labeling at a C-terminal residue, where structural fraying is common. Next, we measured the distance distributions between a pair of spin labels using the DEER experiment. Singly-labeled PA constructs should exhibit a DEER oscillation only if the peptides form dimers or higher oligomers. DEER traces on 150 μM PA-12 (Figures 2A,B, and S8) exhibited a modulation with a depth of 4%, indicating that the spin labels are sufficiently close to exhibit a pronounced dipolar coupling (i.e., about 1.8–6 nm apart). Analysis of the DEER traces yielded a distance distribution with a most probable distance of 3.3 nm, in agreement with the AlphaFold2 model of the (HhH)_2_-Fold dimer (Figure 2C). Reducing the peptide concentration led to reduction in modulation depth without altering the distance distribution (Figure S9A–C). From a series of dilutions and the associated modulation depths^36,37^ (Figure S10A–C), we calculated the dimer dissociation constant (*K*_d_) to be 233 ± 134 μM following eq 1.
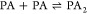
1where PA is a free peptide (monomer) and PA_2_ is the dimer. The large error in *K*_d_ is due to an insufficient number of data points at high concentrations, where solubility is poor. The terminal nitroxide-labeled construct, PA-29, shows a similar modulation depth (Figure 2B), though with a broader distance distribution (Figure 2C), as expected. All DEER experiments were carried out in a MES-buffered solution of D_2_O and in the presence of 20% (v/v) glycerol-*d*_8_ to ensure the formation of a good glass upon freezing and extend the phase memory time. By varying the amount of added glycerol-*d*_8_ and the D_2_O content, we confirmed that neither compound promotes the dimerization of PA-12 (Figures S11 and S12). DEER analysis of the SA-12 peptide, in contrast, did not show any modulation (Figure S8), indicating the absence of dimers in solution.

### PA Has Transient α-Helical Character

To detect α-helical character within PA, we introduced spin labels at positions 2 and 12, which span the first α-helix of the HhH motif (construct PA-2/12; Figure 2A). As PA can form a dimer in solution, we expect to see a superposition of two intra- (2:12, 2′:12′) and four inter-monomer distances (2:2′, 12:12′, 2′:12, 2:12′) or assuming the structure has rotational symmetry as in the AlphaFold2 model, one intra- and three inter-monomer distances. To reduce the contribution of inter-monomer distances, we used a spin-diluted sample comprising a mixture of labeled PA-2/12 and unlabeled PA. Previously, using PA-12 as a model, we found that at about 10% spin-labeled peptide, inter-monomer distances practically disappear, leaving only a background decay (Figure S13A,B). Thus, we performed the DEER measurements on PA-2/12 (Figure 2D,E and S13C) under the same conditions. Although the low spin concentration reduced the signal to noise ratio (SNR), a distance distribution could still be obtained and it exhibited two maxima, one at 2.6 nm and one at 3.3 nm. The former agrees well with an α-helix conformation, which has a distance of 2.45 nm in the AlphaFold2 model as calculated by mtsslSuite. The latter is similar to the 12–12′ dimer distance mentioned above (Figure 2C), although the spin dilution should have suppressed contributions from inter-monomer interactions. To ensure that the 3.3 nm distance does not arise from inter-monomer interactions, we carried out additional DEER measurements on a spin dilution series (Figure S13D–F) and observed that upon going from 10% labeled protein (350 μM total) to 5% labeled protein (400 μM total), the modulation depth did not change and the 3.3 nm peak did not disappear. This result is inconsistent with an inter-monomer interaction. Perhaps the 3.3 nm distance relates to an unfolded or frayed peptide? To test for this possibility, we performed DEER of PA-2/12 under denaturing conditions in 3 M guanidium hydrochloride (Figures 2D and S13C). The resulting distance distribution lacked the peak at 2.6 nm and instead had a broad distance distribution centered at 3.3 nm (Figure 2E). Consequently, the longer distance associated with PA-2/12 likely corresponds to an unfolded α-helix and a potentially open structure.

Given the high labeling efficiency (>95%), the modulation depth of PA-2/12 at 90% spin dilution (where intra-monomer interactions should dominate) was lower than the expected Δ_max_ = 13 ± 3 under these W-band DEER experimental conditions. Likewise, the modulation depth of the denatured sample was only 2.7%, also significantly lower than expected. These low modulation depths may be an indication of dipolar couplings outside the detection window of the DEER.

Given the marginal stability of the dimer state, the key question now becomes whether the conformational states we have demonstrated in aqueous buffer are accessible in the context of a peptide-RNA coacervate.

### Peering into the PA/RNA Composite

**Figure 3:**
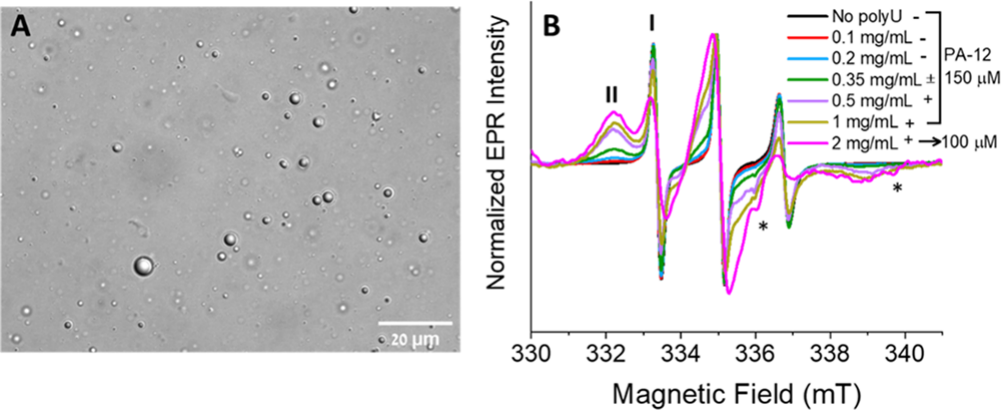
(A) Optical microscopy image of 150 μM PA-12 in the presence of 1 mg/mL polyU. (B) X-band CW-EPR spectra of 150 μM PA-12 recorded at room temperature as a function of the added polyU concentration in mg/mL. The EPR spectrum in magenta is of 100 μM PA-12 in the presence of 2 mg/mL polyU. The presence of fast and slow-motion components is indicated by **I** and **II**, respectively. Plus (+) and minus (−) signs indicate whether droplets were clearly observed under the optical microscope or not, respectively. The “±” sign indicates the presence of very tiny or very few droplets. * denotes cavity background signals.

After characterizing the dynamic and structural properties of PA in solution, we proceeded to explore these properties upon interaction with the homopolymeric RNA molecule polyU. Here, we explore peptide/RNA composites that lead to the formation of coacervates and those that do not. PA-12, like the unlabeled peptide, produced coacervates.^17^ Mixtures of 150 μM PA-12 and 1 or 2 mg/mL polyU readily form coacervates detected by optical microscopy (Figure 3A), whereas only a few tiny droplets were observed at 0.35 or 0.5 mg/mL polyU (Figure S14A,B). Using CW-EPR, binding of PA to RNA can be tracked through spin label dynamics. We initially kept the concentration of PA-12 constant at 150 μM and varied the amount of polyU. The CW-EPR spectra in Figure 3B indicate superposition of two populations, **I** and **II**. While **I** is typical of spins undergoing fast motion, as was observed for unbound PA in solution (either monomeric or dimeric), **II** corresponds to highly restricted rotational diffusion. The relative intensity of **II** increases with the amount of polyU added, indicating binding of PA-12 to polyU. Simulations of the EPR spectra and the associated parameters are given in Table S1 and Figure S15. The percentage of **II** increased from 32% at 0.1 mg/mL polyU to 93% at 1 mg/mL polyU. Increasing to 2 mg/mL polyU and reducing the PA-12 concentration to 100 μM increased the population of **II** to 95% (Figures 3B and S15G). Such restricted motion and dynamic components have also been reported for proteins undergoing phase separation.^38,39^ Mixing unlabeled scrambled peptide with 1 mg/mL polyU, in contrast, did not form coacervates but rather aggregates, as previously reported.^17^

**Figure 4:**
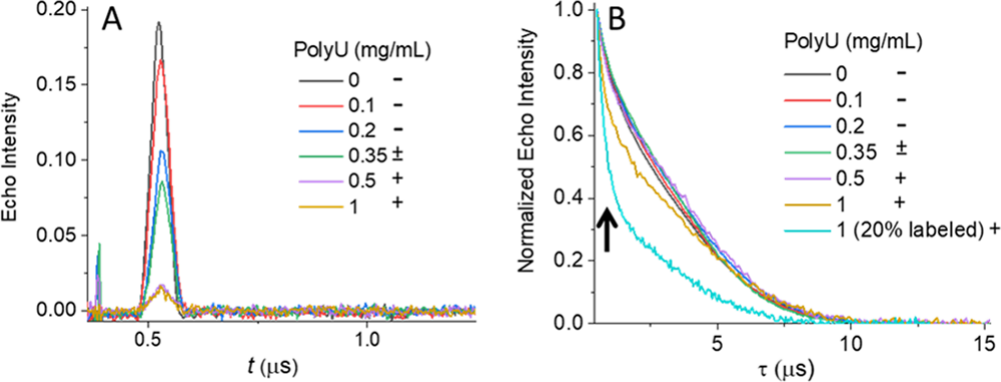
(A) Echo signal and (B) echo-decay curves of 150 μM PA-12 in the presence of different concentrations of polyU. The echo decay in cyan contains 20% spin-labeled PA-12 and is associated with a fast-decaying component (denoted by an arrow) that is suppressed in the undiluted sample. + and – indicate whether droplets are clearly observed under the optical microscope or not, respectively; “±” indicates the presence of very tiny or very few droplets.

**Figure 5:**
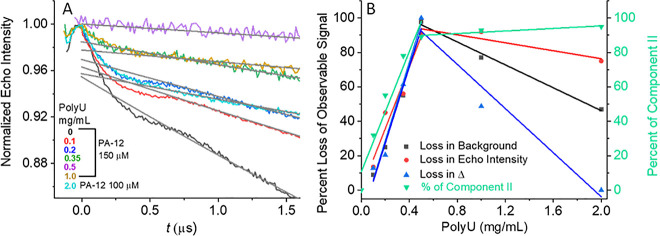
(A) Primary DEER traces of PA-12 in the presence of different polyU concentrations. For samples with 2 mg/mL polyU, the PA-12 concentration was 100 μM. The thin gray lines show the background decay of the DEER trace. The state of the samples in terms of droplet formation is the same as in Figure 4. (B) Summary of loss in background decay (black), loss in echo intensity (red), and loss in modulation depth, Δ (blue), (left *Y*-axis) and comparison with relative abundance of population **II** (green) from the simulation of CW-EPR spectra in Figure 3B (right *Y*-axis). The signal loss was calculated relative to the 150 μM sample with no added polyU.

The CW-EPR spectra reveal that PA binds to polyU in a manner that restricts its motion significantly. However, to obtain structural information, we need to proceed with DEER measurements. In coacervates, the local concentration of PA is anticipated to be significantly higher than in solution. The sensitivity of CW-EPR and pulse EPR (e.g., DEER) to high local spin concentrations, however, is fundamentally different: CW-EPR can tolerate high spin concentrations because it is less sensitive to phase relaxation, which increases significantly with increasing spin–spin interaction, and therefore, all spins contribute to the signal. DEER measurements (and pulse measurements in general), on the other hand, are highly sensitive to fast phase relaxation and therefore at high local spin concentrations, a significant fraction of the spins escape detection. Accordingly, before proceeding to DEER measurements, we explored the effect of added polyU on the echo intensity and the echo decay rate, which is a measure of phase relaxation. Using the samples from the above mentioned series (150 μM PA-12 mixed with varying concentrations of polyU; Figure 3), we measured the echo intensity, which corresponds to the number of observable spins in the samples. A significant decay in echo intensity occured with increasing concentrations of polyU (Figure 4A), ultimately reaching a plateau at 0.5 mg/mL polyU, where only 8% of the signal remained. We also found a close correlation between population **II** from the CW-EPR spectra and the loss of echo intensity as a function of polyU concentration (Figure 5B). Taken together, we conclude that the local concentration of PA-12-bound to polyU is high.

Next, we carried out echo decay measurements in an attempt to resolve the different contributions of population **I** and **II** to the observed echo signal. We found that the normalized echo decay curves at low concentrations of polyU, ranging from 0 to 0.5 mg/mL, were very similar (Figure 4B), although the echo intensity was significantly reduced, suggesting that unbound peptide (population **I**) is the primary source of observable spins at these concentrations. As the polyU concentration increases, this fraction noticeably decreases. However, a small difference in echo decay is observed when the polyU concentration exceeds 0.5 mg/mL, and a fast-relaxing component becomes evident (Figure 4B). The appearance of a fast-relaxing component suggests that some signal is being regenerated at high concentrations of polyU, possibly due to a dilution of the PA-12 molecules bound to polyU. To confirm this interpretation, we carried out echo decay measurements on a sample with 1 mg/mL polyU and 80% spin-dilution (final PA concentration 150 μM). This dilution should reduce the local spin concentration of polyU-bound PA. For this sample, the fast-decaying component became clearly visible (Figure 4B), confirming that at 0.5 mg/mL polyU and below, the high local concentrations of PA-12 bound to polyU suppresses the echo intensity. However, above 1 mg/mL polyU, the spacing between PA-12 molecules becomes sufficiently large for some spin echo detection. The implication of these results is that high concentrations of polyU may be amenable to distance measurements by DEER analysis. DEER measurements made with 2 mg/mL polyU confirm this interpretation, as discussed in greater detail in the next section.

### PA Dimer Detected within Peptide-RNA Coacervates

We carried out DEER measurements on the series of samples with 150 μM PA-12 and varying amounts of polyU (Figure 5A). DEER traces were analyzed in terms of several parameters: (i) the modulation depth, Δ, which gives information about the amount of dimers present; (ii) the slope of the background decay, which gives information on the average local concentration of the observed spins;^36,40^ and (iii) the SNR, which indicates how many spins were observed. The DEER traces show that as the polyU concentration was increased, the modulation depth, the SNR, and the slope of the background decay decreased. At 0.5 mg/mL polyU, modulation could no longer be detected. The decrease in the background decay slope and SNR are consistent with the observed reduction in echo intensity (Figure 4A). We therefore conclude that the observed DEER modulation arose from the unbound PA-12 dimers, whereas the polyU-bound PA-12 could not be detected, as discussed in the description of the echo decays. As the polyU concentration increases, the PA-12 concentration in the dispersed phase decreases and therefore the dimer population decreases as well (eq 1), ultimately resulting in the disappearance of the modulation at about 0.5 mg/mL polyU.

Interestingly, modulation reappeared again upon increasing the polyU concentration to 1 mg/mL (Figure 5A, yellow), which is also when the presence of coacervates becomes clear (Figure 3). The appearance of a fast-relaxing component in the echo decay and DEER modulation indicates the presence of contributions from polyU-bound PA-12 molecules in liquid condensates and demonstrates that dimers exist within the coacervates. To further confirm that increasing the relative amount of polyU increases the average distance between bound PA molecules, thereby allowing a larger fraction of spins to be observed, we increased the polyU concentration to 2 mg/mL and decreased PA-12 concentration to 100 μM (Figure 5A). As expected, this sample showed higher modulation depth than the sample with 1 mg/mL polyU.

We summarized our experimental observations in Figure 5B, where we plot the decrease degree (in %) in the background decay, the echo intensity, and the modulation depth as a function of polyU concentration. For all parameters, the graphs show a similar linear behavior in the high PA/polyU regime below 0.5 mg/mL polyU. Interestingly, the loss of these signals correlates with the percent increase in population **II** (RNA-bound PA) as detected in the CW-EPR spectra. At 0.5 mg/mL polyU, an inflection point is detected: Above 0.5 mg/mL polyU, population **II** reaches a plateau and we have a small gain in echo intensity and background decay, but a large gain in modulation depth. From this series of experiments, we conclude that PA bound to polyU has restricted motion and that dimers of PA exist also when bound to polyU within the coacervate. We determined the local concentration of the spins contributing to the DEER traces in the PA/polyU mixtures from a calibration curve generated from the background decay slopes of the DEER data of PA-12 in the absence of polyU (Figure S10D), and the results are given in Table 2. It clearly shows how the local concentration decreases with added polyU up to PA-12/polyU 150 μM/0.5 mg/mL, reaching a value of 2.3 μM and then it increased to 23 μM for PA-12/polyU of 150 μM/1 mg/mL. This increase is also associated with an increase of the modulation depth to 2%, which is higher than the value expected from a solution concentration of 23 μM Figure S9B), suggesting that binding to polyU promotes dimerization. We now consider the complementary titration, where the polyU concentration is held constant and the concentration of PA is varied.

**Table 2:** Local Concentrations of the Detected Spins Determined from the DEER Background Decay for the Various PA-12/polyU Samples

PA-12 (μM)/polyU (mg/mL)	local concentration (μM)
150/0.1	91 ± 8
150/0.2	75 ± 7
150/0.35	45 ± 4
150/0.5	2.3 ± 2
150/1	23 ± 2
100/2	53 ± 5
100/1	48 ± 4.5
30/1	25 ± 2

### RNA Binding Promotes HhH Dimerization

**Figure 6:**
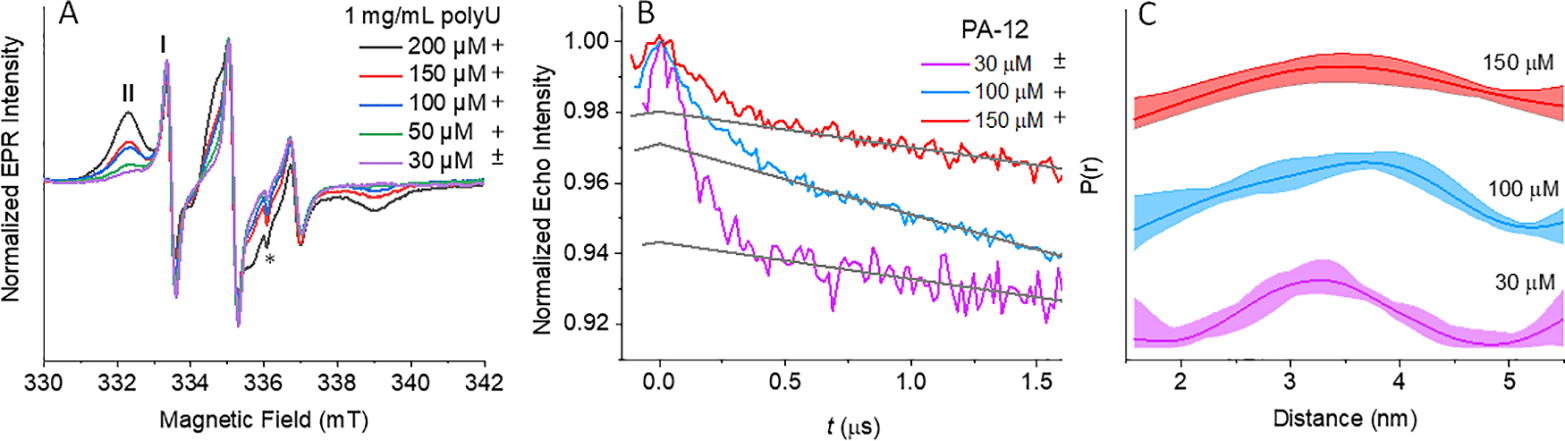
(A) X-band CW-EPR spectra of 30–200 μM PA-12 in the presence of 1 mg/mL polyU. The presence of fast and slow-motion components are indicated by I and II.' * denotes the cavity background signal. (B) Primary DEER trace with background decays indicated in gray. (C) Distance distribution of 30, 100, and 150 μM PA-12 in the presence of 1 mg/mL polyU. The + and – symbols indicate whether droplets are clearly observed under an optical microscope or not, respectively, and the “±” symbol indicates the presence of very tiny or very few droplets.

In the titrations mentioned above, the percent of PA dimers in solution was significant due to the relatively high concentrations of PA (100–200 μM). Here, we maintain the polyU concentration at 1 mg/mL, while changing the PA concentration, starting from a region where the percent of dimers in solution is very low. Under the optical microscope, we observed a significant number of droplets at concentrations at 50 μM PA-12 (Figure S14C,D) and above. The CW-EPR spectra, given in Figure 6A, reveal an increase of the slow-motion component **II** concomitant with an increase in PA-12 concentration. The simulation of the spectra showed that the fractional population of **II** increased from 60% at 30 μM PA-12 to 96% at 200 μM PA-12 (Figure S16 and Table S2).

DEER measurements on 30, 100, and 150 μM PA-12 in the presence of 1 mg/mL polyU are shown in Figure 6B. The DEER traces show the highest modulation depth for 30 μM PA-12, consistent with a minimum loss in echo intensity and the highest fraction of observable spins (Figure S17A). Surprisingly, the modulation depth in the presence of polyU is 5.7%, for a local concentration of 25 μM (see Table 2), which is significantly higher than the 2% for 30 μM solutions of PA-12 without polyU (Figure S17B,C). This result clearly shows that binding of PA to polyU promotes dimerization as suggested above. The CW-EPR indicated that this sample consists of 40% of component **I**, earlier assigned to free PA; this yields a solution concentration of 12 μM, which should consist primarily of monomeric PA, therefore reducing the maximum possible modulation depth by 40%. The resulting maximum modulation depth is 9.5%, in agreement with our experimental conditions for 100% spin pairs (Δ_max_ = 13 ± 3). This observation also implies that the majority of observed bound PA is in the dimeric form. At 100 and 150 μM of PA, where bound PA comprises 90 and 93% of PA molecules, the modulation depth was lower, namely, fewer dimers were observed than at 30 μM, although the local concentration was either higher or similar. We attribute this reduction to the higher fraction of observed spins that contribute more inter-molecular distances, which broadens the distance distribution (Figure 6C) and can be outside the DEER detection window and/or to the presence of a higher fraction of bound monomeric PA.

In the absence of polyU, we confirmed the presence of the α-helical structure in PA-2/12 for a relatively minor population. We also checked if binding of polyU results in any alteration of the α-helical structure. We carried out DEER measurements on PA-2/12/polyU 100 μM/2 mg/mL, which is in the coacervate forming regime, and with 20% of the peptides spin-labeled (Figure S18). We observed a difference in the width of the distance distribution that could be a consequence of inter-monomer contributions, considering that we have used only 1:5 dilution. Therefore, we cannot derive concrete conclusions regarding the structural changes induced by binding. We also attempted spin dilution of the 30 μM peptide and 1 mg/mL polyU condition, where we observed the highest modulation depth for polyU-bound PA (Figure 6B), but the SNR was too low to draw any concrete conclusions.

## Discussion

### Coacervate Formation by PA: A Microscopic View

What prime processes describe the formation of coacervates by PA? We will attempt to answer this question by accounting for all EPR results combined with the optical microscopy observation of coacervates. In doing so, we will construct a consistent picture of the species present in solution as a function of PA/polyU ratio and their relation to the formation of coacervates. In the absence of RNA, PA forms dimers in solution with a *K*_d_ of 233 ± 134 μM, and the potential for dimer formation of PA was confirmed by MD simulations (Figure S6). We now postulate several prime processes coexisting in solution, in addition to the solution dimerization of PA (eq 1, above).

**Figure 7:**
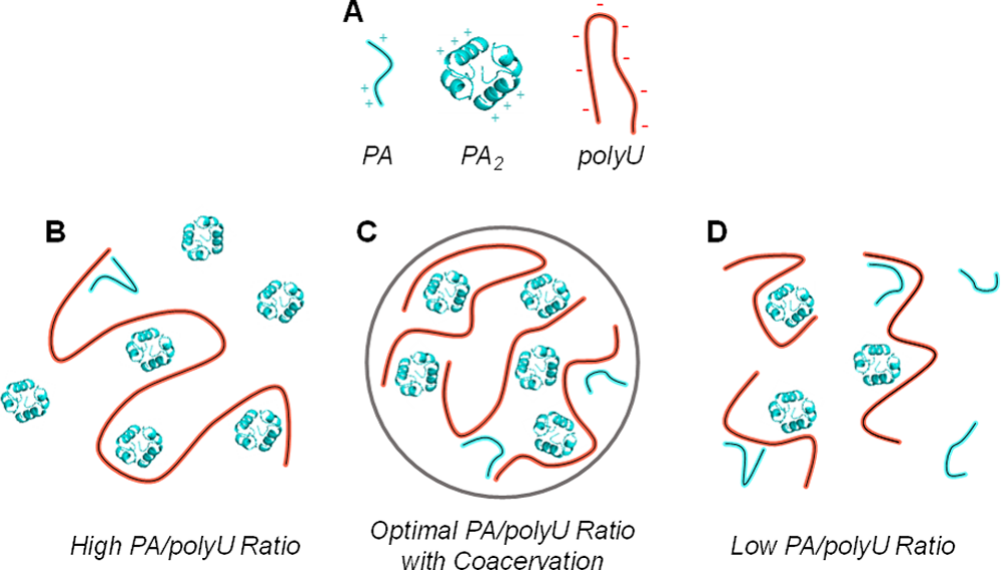
Schematic representation of (A) polyU, dimer and monomer of PA, (B) high PA/polyU regime with no coacervates, (C) coacervate formation via cross-linking with optimum PA/polyU ratio, and (D) low PA/polyU regime with no coacervate formation.

As one molecule of polyU has multiple negatively charged binding sites (Figure 7A), it can bind to a number of PA molecules, be it PA (the monomer) or PA_2_ (the dimer). Moreover, considering their positive charges, single PA or PA_2_ molecules can bind to one site on polyU or two sites on the same polyU molecule, forming intra polyU cross-links. Finally, they can bind to two sites on two different polyU molecules, leading to inter-polyU cross-linking. MD simulation of PA dimers in the presence of two molecules of 20 nucleotide polyU supports a model in which the dimeric structure has at least two binding sites to RNA, with each site possessing seven arginine residues and therefore an extensive interface with RNA (Figure S6, panel D). The putative binding mode is distinct from that observed in dsDNA binding, which is centered on the PGIGP binding loop.^41^ We assume that the likelihood of PA dimerization occurring on the RNA is a comparably slow process, owing to its restricted diffusion. Dimerization can take place during the off state, where two PA molecules are brought closer as a consequence of binding to polyU.

When discussing the interaction of PA with polyU, we consider three regions of composition. The first is high PA/polyU regime (Figure 7B), where the dimer concentration is high such that PA_2_ is predominates and it can bind to one or two sites on the polyU. Furthermore, under this condition, not all PA molecules are bound to polyU. Nonetheless, because of the excess PA, and consequently the population of PA_2_ coordinated in a bidentate mode, intra-polyU crosslinks are preferentially formed. This binding of either PA or PA_2_ to polyU leads to a restricted rotational diffusion and corresponds to population **II**, which exhibits the slow-motion CW-EPR spectrum. In addition, the bound PA_2_ has a high local concentration and therefore its echo decays very fast (Figure 4) and does not contribute to the DEER signal. Accordingly, the DEER signal oscillations are generated by the free PA_2_ in solution (Figure 5A). We cannot exclude the possibility that some PA binds to the polyU and contribute to component **II** as well; however, given that high concentrations of PA promote dimerization, and that the dimer is further stabilized upon polyU binding, we think that PA binding is less likely than PA_2_ binding. Under these conditions, there are no coacervates because inter-polyU interactions are scarce. As the polyU concentration increases, more PA_2_ molecules bind, the concentration of free PA_2_ decreases, the DEER modulation depth decreases, and inter-polyU cross-linking takes place, as shown in Figure 7C. In this regime of intermediate PA/polyU, where inter-polyU cross-linking becomes significant, coacervates are formed. Decreasing the PA/polyU ratio reduces the local concentration of bound PA_2_, thereby leading to the reappearance of modulation in the DEER traces.

Finally, we consider the low PA/PolyU regime (Figure 7D). Interestingly, the DEER trace of 30 μM PA in 1 mg/mL polyU (which barely forms coacervates) supports binding of PA_2_, while the CW-EPR spectrum shows the presence of 40% component **I**. This can be explained by two scenarios; one is that monomeric PA has a lower preference to RNA, so it remains in solution and is responsible for the fast motion component. This option relies on the dimer having conformations where positive and negative charges are separated, allowing for efficient bidentate binding to the polyU. The model shown in Figure 1B is consistent with this option. Another possibility is that PA binds to RNA with only one anchoring point and retains high mobility similar to that of unbound peptide. This possibility is consistent with a recent EPR study on a truncated fraction of tau that forms coacervates in the presence of polyU at low salt concentrations; although, unlike with PA, no change in dynamics was observed in either the absence or presence of polyU.^42^ Tau has also been shown to form coacervates at high salt concentration (3.75–4.75 M) without RNA, and in this case, the slow-motion population was assigned to liquid–liquid phase separation-driven aggregate formation.^43^ We find this option less likely because there are regions of PA/polyU where almost all PA is under restricted motion (100 μM PA-12/2 mg/mL polyU) and it is unlikely that all PA molecules—monomer or dimer—are bidentate-bound.

The effect of PA_2_/PA binding to polyU and its effect on the inter- and intra-molecular polyU associations can also be discussed in terms of charge distribution. In the low PA/polyU regime, the excess negative charge of polyU leads to charge repulsion and prevents inter-molecular cross-linking. In the high PA/polyU regime, polyU molecules could be saturated with bound PA_2_/PA, and this in turn can lead to charge inversion of RNA due to increasing positive charges of the bound PA_2_/PA over the negative charge of polyU, reducing the possibility of inter polyU cross-linking again owing to electrostatic repulsion. Such inversion of the charge of RNA or DNA in the presence of small polycationic species has been shown to be responsible for reentrant phase behavior.^44−46^ Only when the PA and polyU charges are matched, does the inter-molecular polyU cross-linking become efficient and lead to the formation of coacervates. Complex coacervation and reentrant behavior have been extensively studied, including systems comprising peptide-RNA, polycation-RNA, and polyamines-nucleic acids.^47,48^ Finally, we note that arginine-rich domains are prone for phase separation in the presence of RNA due to electrostatic interaction^49^ and the ability of the arginine side chain to simultaneously form a higher number of specific interactions with oligonucleotides.^50^ However, the SA peptide, which has the same number of arginine residues, and at the same positions, did not form coacervates with polyU, suggesting that the sequence (and not just composition) is crucial for dimer formation and the generation of transient structure for phase separation. Although we suspect that attainment of the α-helical structure is coupled to dimerization, this was not explicitly shown.

### Coacervates as a Cradle for Protein Evolution

Previously, the PA peptide was demonstrated to have a weak α-helical propensity and form coacervates with polyU, but no indications of dimerization were detected.^17^ Nevertheless, duplication and fusion of the PA peptide resulted in an α-helical dsDNA binding domain that likely adopts the (HhH)_2_-Fold.^17^ We now report the observation of PA dimers and evidence of transient α-helical folding in aqueous buffer. These results agree well with the growing appreciation that oligomers are a key intermediate in the evolution of structured domains, particularly of symmetric and repetitive protein architectures.^9−14^ The stability of the dimer, however, is modest (*K*_d_ of 233 ± 134 μM), indicating that the PA peptide exists *on the cusp of foldability*. The PA peptide, therefore, represents an evolutionary intermediate just prior to the emergence of an independently folding protein domain. Consequently, we expect PA dimerization to be sensitive to the surrounding environment—as is common for simplified proteins and peptides^35,51,52^—making it an ideal model system to study protein structure evolution in the coacervate context.

**Figure 8:**
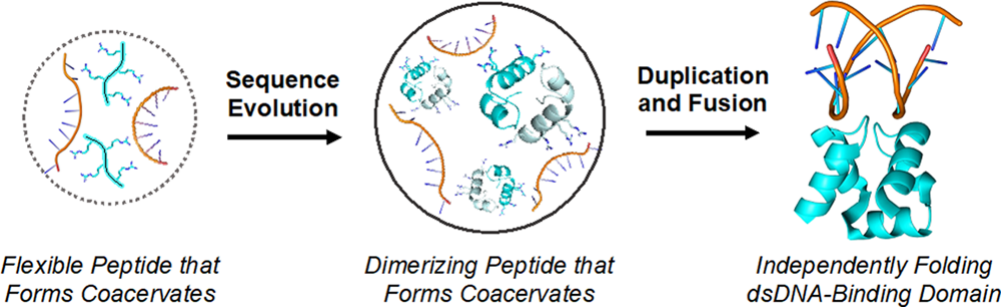
Evolutionary continuity between a simple, coacervate-forming polypeptide (left) and a folded dsDNA-binding domain (right; PDB code 1c7y) via a dimerizing, coacervate-associated intermediate (middle; see also Figure 7C).

A central challenge in the field of protein evolution is to understand how the complex structures of contemporary biology could have emerged “from so simple a beginning.” It has been previously argued that independently folding nucleic acid-binding domains, such as the (HhH)_2_-Fold, may be evolutionarily continuous with simple, flexible peptides that form coacervates with RNA (Figure 8).^6,17^ The key intermediate in this hypothetical trajectory is a coacervate formed by a partially folded peptide that interacts with RNA. This intermediate bridges coacervates formed by flexible, compositional peptides interacting with RNA^53^ and folded dsDNA-binding domains. The observation that PA dimers are promoted by binding to RNA and persist within (and potentially dominate) peptide-RNA coacervates supports this evolutionary model. We conclude that peptide-RNA coacervates were a potential resource for the evolution of structured domains and that the coacervate context can serve as a unique testing ground for novel oligomeric states, which are later capitalized on by common evolutionary processes such as duplication and fusion.^20^

## Conclusions

In this work, we explored the structure of an ancient nucleic acid binding motif alone, bound to RNA, and within coacervates. Using a combination of state-of-the-art EPR spectroscopic techniques complemented by MD simulations, we show that dimerization of the flexible PA peptide is promoted upon binding to RNA, and that dimers are enriched in coacervates. Distance measurements between spin labels revealed that the dimer structure is consistent with the symmetric (HhH)_2_-Fold, which in contemporary biology binds to the minor groove of dsDNA but in coacervates serves to form bridging bidentate interactions between molecules of polyU. These observations demonstrate how structural and functional plasticity allow for a continuous transition from a flexible, coacervate-forming peptide to a stable, structured protein domain with a related but distinct function. Finally, we probe the process of coacervation itself and describe how the ratio of PA to polyU tunes the extent of coacervation by altering the prevalence of cross-linking.

## Supporting Information Available

Characterization of the spin-labeled peptides, echo-detected EPR spectrum, MD simulations data, EPR and DEER data of PA-12 and PA-2/12 in solution, determination of the dimerization *K*_d_, effect of D_2_O and glycerol, spin dilution experiments, microscope images, CW-EPR simulation of PA-12/RNA mixtures, and echo intensity and DEER data of PA-12 with and without polyU (PDF)
