# Correction to “Peptide-RNA Coacervates as a Cradle for the Evolution of Folded Domains”

**DOI:** 10.1021/jacs.2c11057

**Published:** 2022-11-03

**Authors:** Manas Seal, Orit Weil-Ktorza, Dragana Despotović, Dan S. Tawfik, Yaakov Levy, Norman Metanis, Liam M. Longo, Daniella Goldfarb

The Precursor-Arg (PA) dimer model shown in Figure 1 of the main text, which was used for predicting distance distributions (Figure 2, main text) and as a starting point for molecular dynamics simulations (Figure S6), was not generated by the ColabFold implementation of AlphaFold2,^1^ as stated in the text, but by the SWISS-MODEL^2^ server (swissmodel.expasy.org). The input sequence was that of Primordial-(HhH)_2_-Arg,^3^ and the template structure was the C-terminal (HhH)_2_-Fold domain from PDB ID 2nrv chain A. To generate a symmetric dimer structure, the first HhH motif of the homology model, which adopts a canonical conformation, was extracted and overlaid onto the second half of the structure. The N-terminal α-helix of each monomer was extended by two residues (Arg1 and Ile2) because these residues were absent from the homology model obtained from the output of SWISS-MODEL. We thank Lars Eicholt for alerting us to the mistake and apologize for the error.
