# Supplementary material for: Peptide-RNA Coacervates as a Cradle for the Evolution of Folded Domains
Source: J Am Chem Soc. 2022 Jul 29;144(31):14150–60. doi: 10.1021/jacs.2c03819 (PMC9376946; doi:10.1021/jacs.2c03819)
Supplement: Supplementary file 1 — ja2c03819_si_001.pdf [file ja2c03819_si_001.pdf]

Supporting Information

Peptide-RNA Coacervates as a Cradle for the Evolution of Folded Domains

Manas Seal[1], Orit Weil-Ktorza[2], Dragana Despotović[3], Dan S. Tawfik[3‡], Yaakov Levy[4], Norman Metanis[2,5,6], Liam M. Longo[7,8*], Daniella Goldfarb[1*]

[1]Department of Chemical and Biological Physics, Weizmann Institute of Science, Rehovot 7610001, Israel.
[2]Institute of Chemistry, Hebrew University of Jerusalem, Jerusalem 9190401, Israel
[3]Department of Biomolecular Science, Weizmann Institute of Science, Rehovot 7610001, Israel.
[4]Department of Chemical and Structural Biology, Weizmann Institute of Science, Rehovot 7610001, Israel.
[5]Casali Center for Applied Chemistry, The Hebrew University of Jerusalem, Jerusalem 9190401 (Israel)
[6]The Center for Nanoscience and Nanotechnology, The Hebrew University of Jerusalem, Jerusalem 9190401 (Israel)
[7]Earth-Life Science Institute, Tokyo Institute of Technology, Tokyo 152-8550, Japan.
[8]Blue Marble Space Institute of Science, Seattle, Washington 98104, United States of America.

*To whom correspondence should be address:
llongo@elsi.jp, daniella.goldfarb@weizmann.ac.il

Contents

1. **Characterization of the spin labeled peptides** S2
2. **Echo detected EPR spectrum** S6
3. **MD simulations** S6
4. **EPR and DEER data of PA-12 and PA-2/12 in solution** S7
5. **Determination of the dimerization K_d** S8
6. **Effect of D_2O and glycerol** S10
7. **Spin dilution experiments** S11
8. **Microscope images** S12
9. **CW-EPR simulation of PA-12/RNA mixtures** S13
10. **Echo intensity and DEER data of PA-12 with and without polyU** S15

‡ Professor Dan S. Tawfik passed away on 4th May, 2021.

1. Characterization of the spin labeled peptides

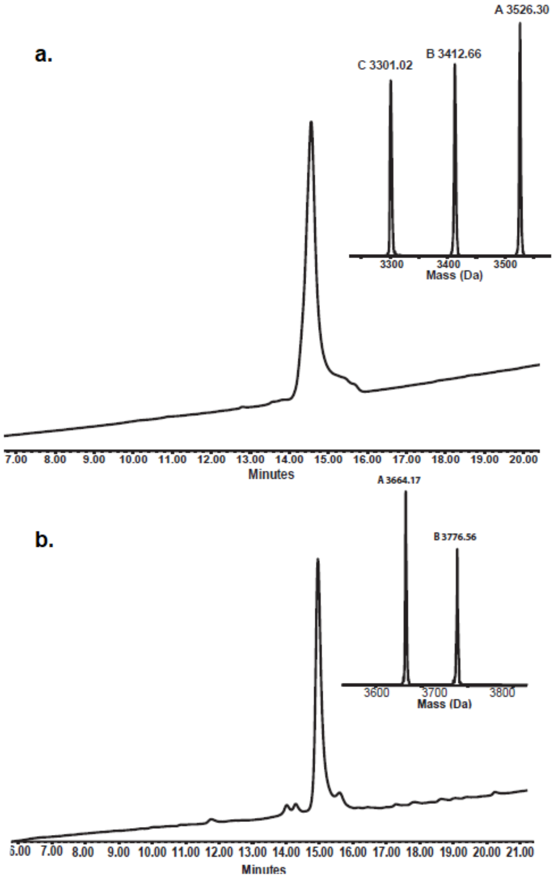

Figure S1. HPLC chromatograms and ESI-MS for purified peptide PA-12, with the inset showing the corresponding mass; **a**. before labeling (calc. 3301.97 Da; obs. 3301.02 Da, [M+TFA] 3412.66 Da, [M+2 TFA] 3526.30 Da). **b**. after labelling reaction (calc.3553.27 Da, [M+TFA] 3667.29 Da; obs. 3664.17 Da, [M+2 TFA] 3776.56 Da).

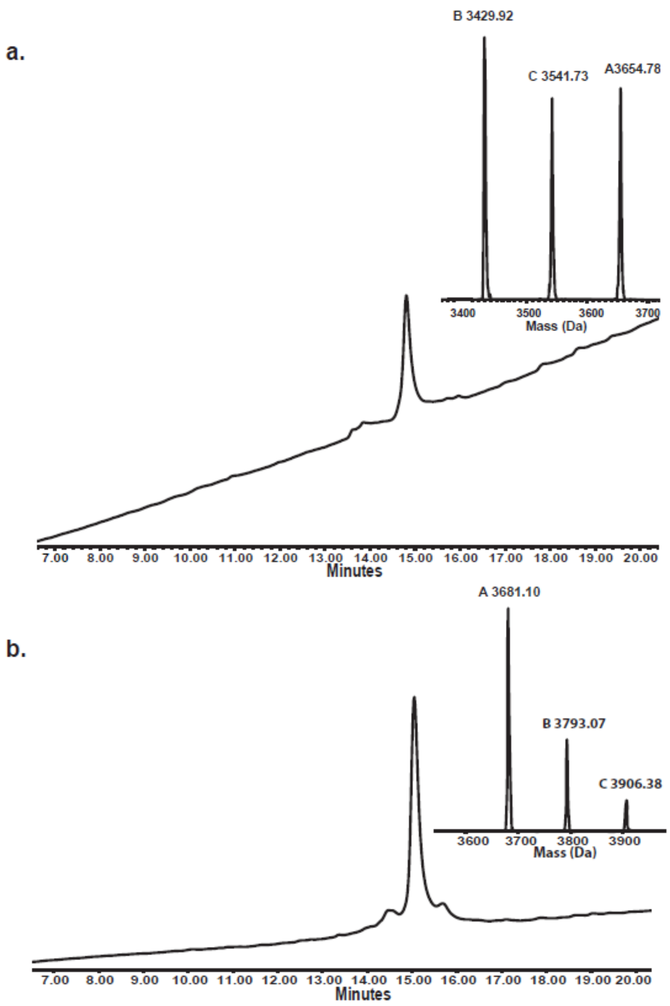

Figure S2. HPLC chromatograms and ESI-MS for purified peptide PA-29, with the inset showing the corresponding mass **a.** before labelling (calc. 3431.08 Da; obs. 3429.92 Da, [M+TFA] 3541.73 Da, [M+2 TFA] 3654.78 Da). **b**. after labelling reaction (calc. 3682.38 Da; obs. 3681.10 Da, [M+TFA] 3793.07 Da, [M+ 2 TFA] 3906.38 Da).

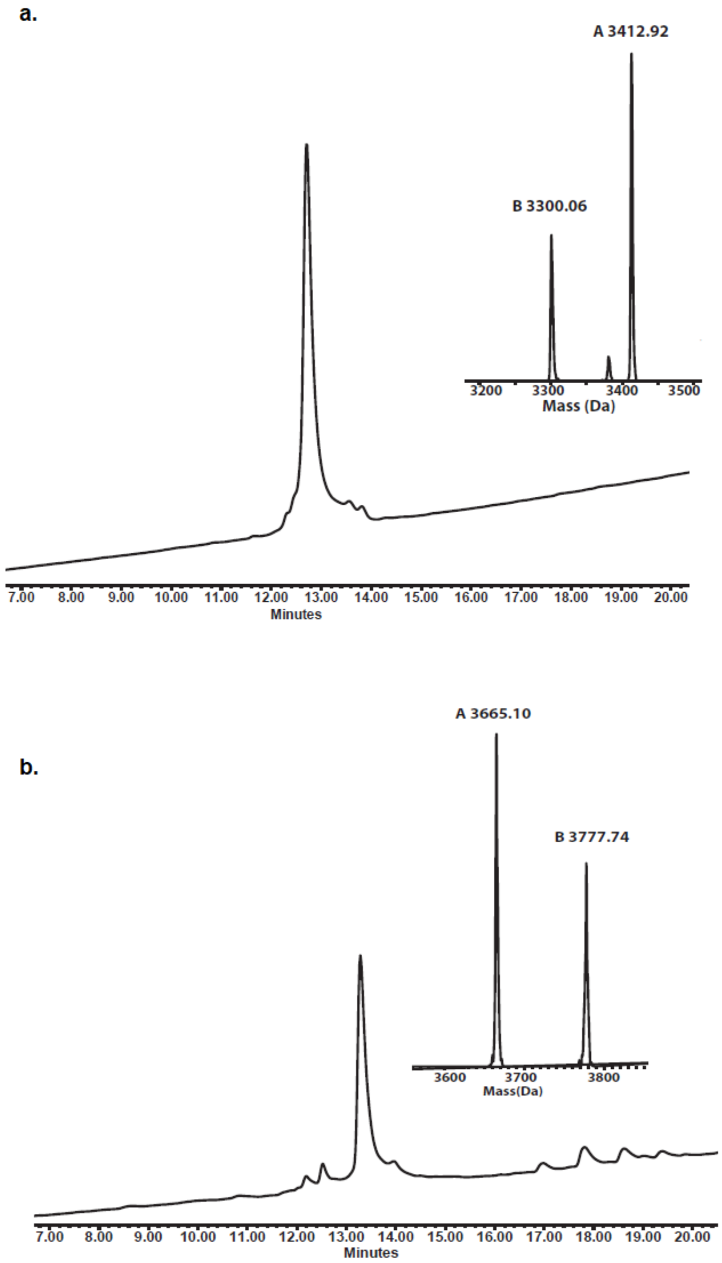

Figure S3. HPLC chromatograms and ESI-MS for purified peptide SA-12, with the inset showing the corresponding mass; **a**. before labelling (calc. 3301.97 Da; obs. 3300.06 Da, [M+TFA] 3412.92 Da). **b**. after labelling reaction (calc.3553.27 Da, [M+TFA] 3667.29 Da; obs. 3665.10 Da, [M+2 TFA] 3777.74 Da).

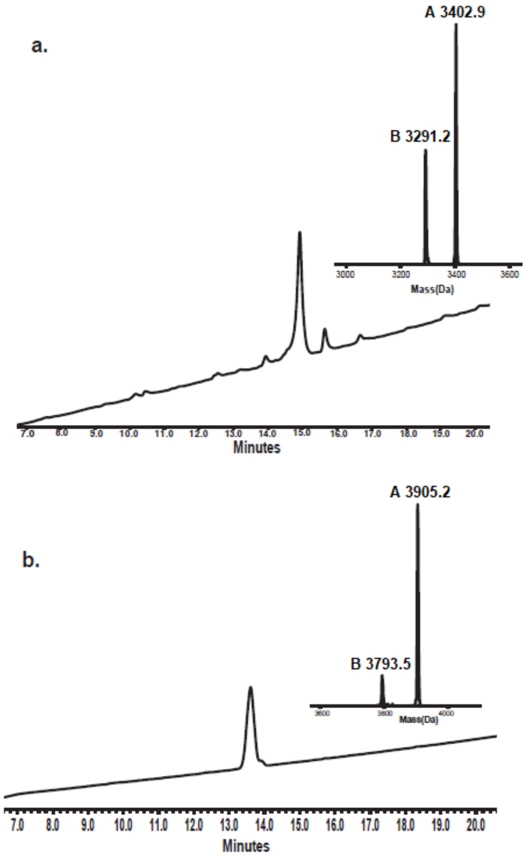

Figure S4. HPLC chromatograms and ESI-MS for purified PA-2/12, with the with the inset showing the corresponding mass **a**. before labelling (calc. 3291.94 Da; obs. 3291.2Da, [M+ 1 TFA] 3402.9 Da); **b**. after labelling reaction (calc. 3794.6 Da; obs. 3793.5 Da, [M+1 TFA] 3905.2 Da).

2. Echo detected EPR spectrum

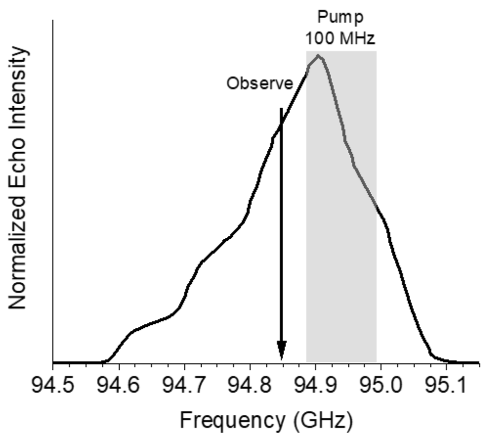

Figure S5. W-band echo detected field sweep spectrum (25 K) of PA-12. The black arrow and shaded area show experimental set up corresponding to observe and pump pulse position in DEER.

3. MD simulations

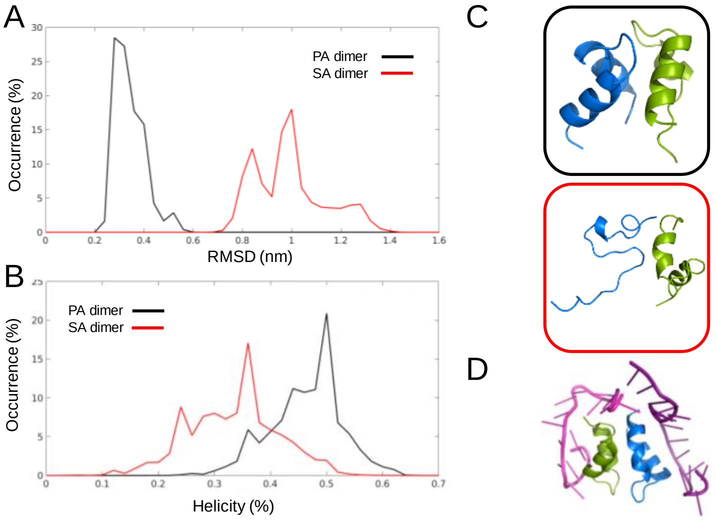

Figure S6. Conformational analysis of the PA and SA dimers using molecular dynamics simulations. A). Distribution of the conformational deviation for the PA (black) and SA (red) sequences modeled following the dimeric structure predicted by AlphaFold2. The conformational deviation is quantified by the mean square distance (RMSD). B). The helical content of the ensemble of conformations of PA and SA sequences sampled in the MD simulations. We note that the helical content of the Alphafold2 predicted structure is 66%. C). Representative conformations of PA and SA sequences (black and red frames,

respectively). D). A representative structure of PA dimer simulated with two strands of polyU, each of 20 nucleotides, illustrating the two positively charged patches on the symmetric dimer.

4. EPR and DEER data of PA-12 and PA-2/12 in solution

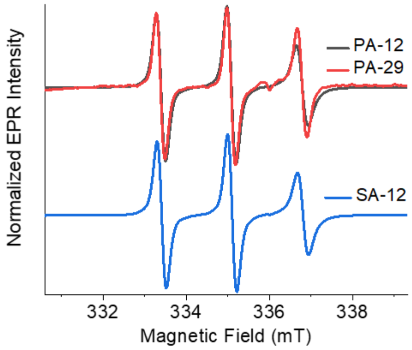

Figure S7. Room temperature X-band CW-EPR spectra of PA-12 and PA-29 and comparison with SA-12 (lower panel).

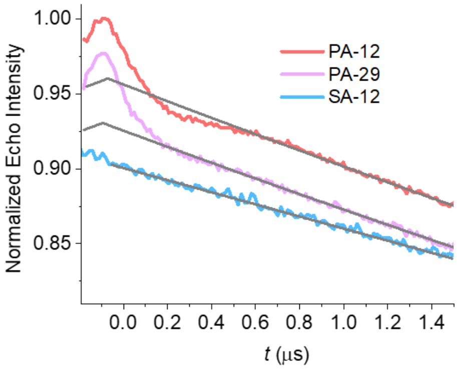

Figure S8. W-band primary DEER data of 150 μM PA-12, 150 μM PA-29 and 150 μM SA-12. The grey line represents the DEER background decay. The traces were vertically shifted for easy comparison.

5. Determination of the dimerization K_d

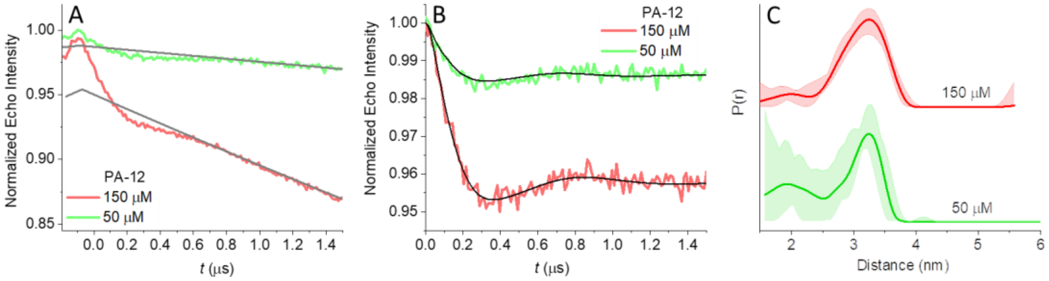

Figure S9. (A) W-band primary DEER data of 150 and 50 μM PA-12 with background decays traces shown in grey. (B) The corresponding background corrected DEER traces and fit in black. C. The corresponding distance distribution. The solid lines represent the distributions with the smallest RMSD in relation to the experimental data; the striped regions indicate the range of alternative distributions (±2 times the standard deviation) obtained by varying the parameters of the background correction and noise, as calculated by the validation tool in the DeerAnalysis software package with the default values.

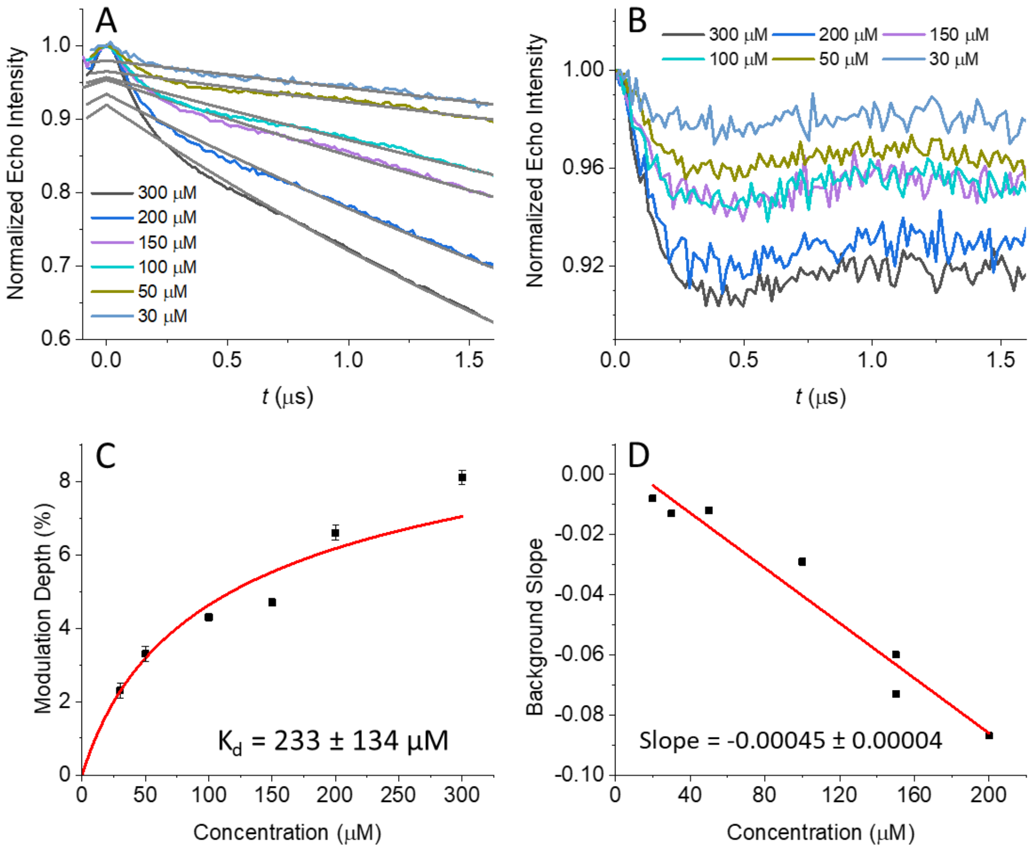

Figure S10. A) Primary and B) background corrected DEER trace of PA-12 at different concentration. The DEER data of the dilution series were measured under different conditions compared to the rest of the DEER data due to the installation of the new microwave amplifier having more power and yielding higher modulation depth. The field was set to the nitroxide maximum intensity at 94.925 GHz, observe pulses were at 94.83 GHz (t_π = 45-50 ns) and pump pulse: 94.88-94.98 GHz (t_p = 128 ns). C) Plot of the modulation depth, Δ, as a function of concentration, *c*. The data was fit to [$\Delta = 0.5\Delta_{max}\{(2+K_d/2c) \pm ((2+K_d/2c)^2-4)^{0.5}\}$] using Origin as previously reported.[1] Due to insufficient data in the high concentration range as a consequence of solubility limitations we fixed Δ_{max} at 13 ± 3 based on estimation of the largest modulation depth at W-band for nitroxide-nitroxide DEER. D) Plot of the DEER background decay slope as a function of the PA-12 concentration in solution, obtained from a different data measured with the old amplifier, so it can be used as a reference for all other data measured with the old amplifier.

6. Effect of D_2O and glycerol

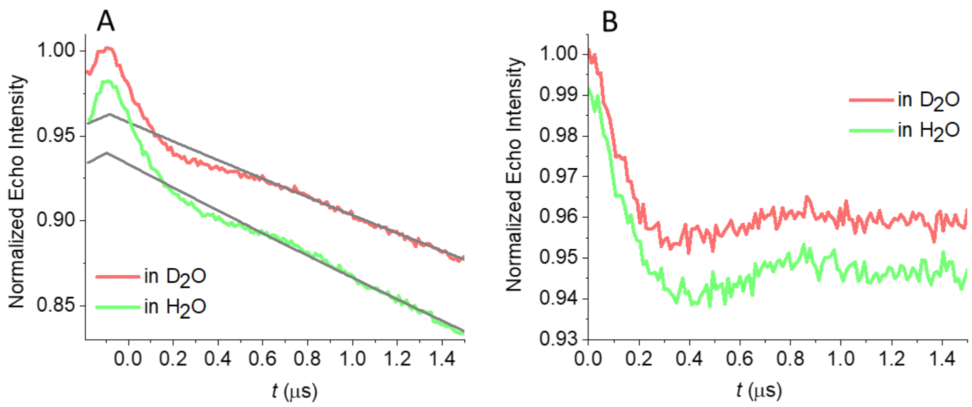

Figure S11. A) W-band primary DEER data of 150 µM PA-12 in D_2O and H_2O in with the background decays shown in grey. B) Background corrected DEER data. The traces are vertically shifted for clear presentation.

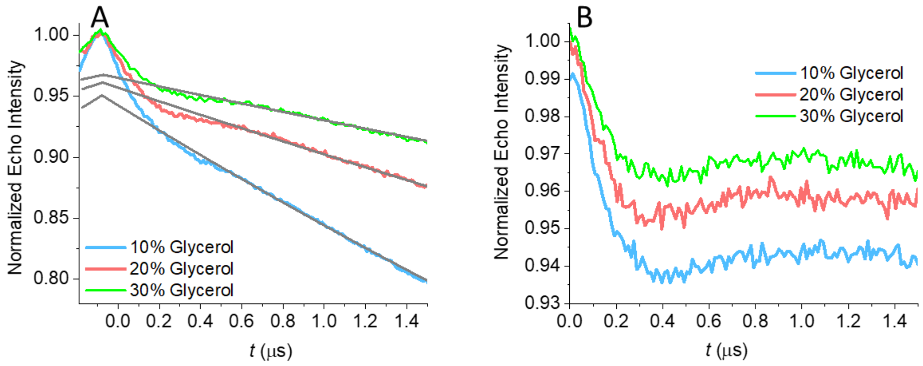

Figure S12. A) W-band primary DEER data of 150 µM PA-12 in 10, 20 and 30% glycerol-d_8 and background decays shown in grey. B) The corresponding background corrected data. The traces in B are vertically shifted for clear presentation.

7. Spin dilution experiments

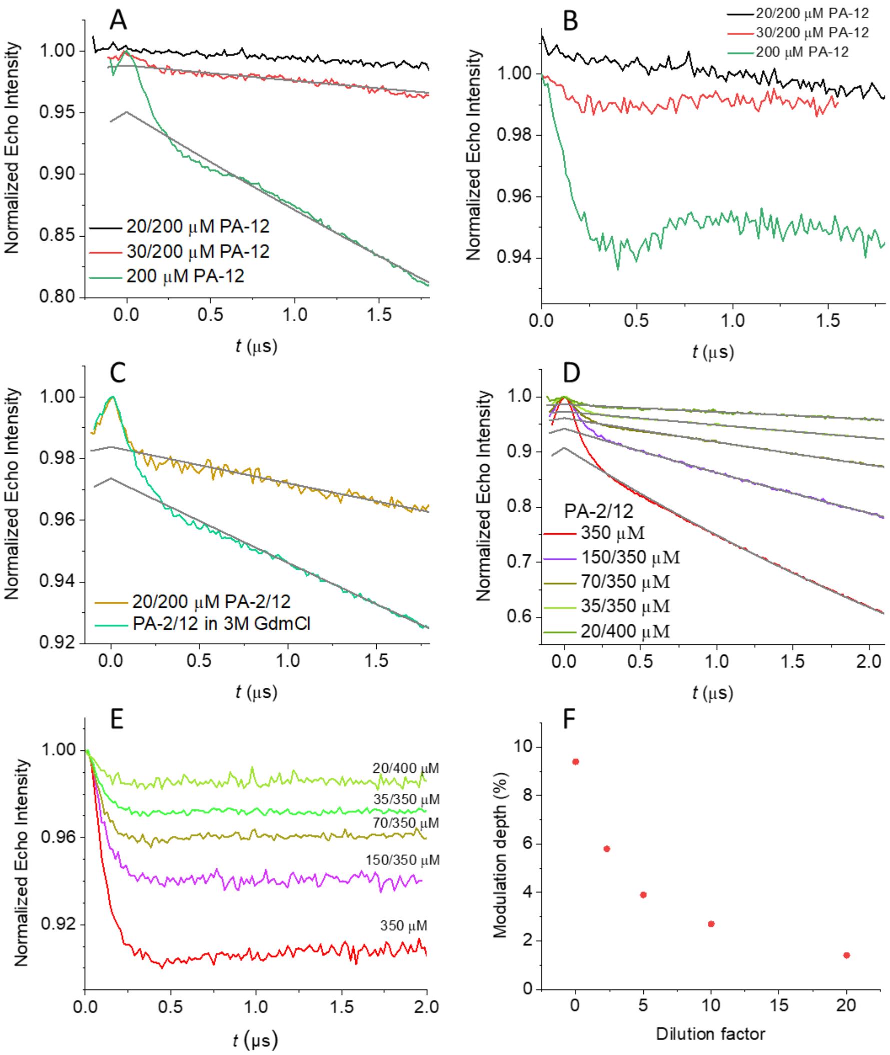

Figure S13. (A) W-band primary DEER trace of PA-12 at with different spin dilution out of total 200 μM PA, where PA-12 concentrations were 20 μM (referred as 20/200) and 30 μM (referred as 30/200). The background decay is given in grey. B) The same data in A after background correction. C) Primary DEER data of PA-2/12 under spin dilution where PA-2/12 concentrations were 20 μM in total PA of 200 μM and 100 μM PA-2/12 in 3M GdmCl. D) Primary DEER data of PA-2/12 under different spin dilution conditions. E) The same data in C after background correction. F) Modulation depth under dilution and spin dilution as obtained from E.

8. Microscope images

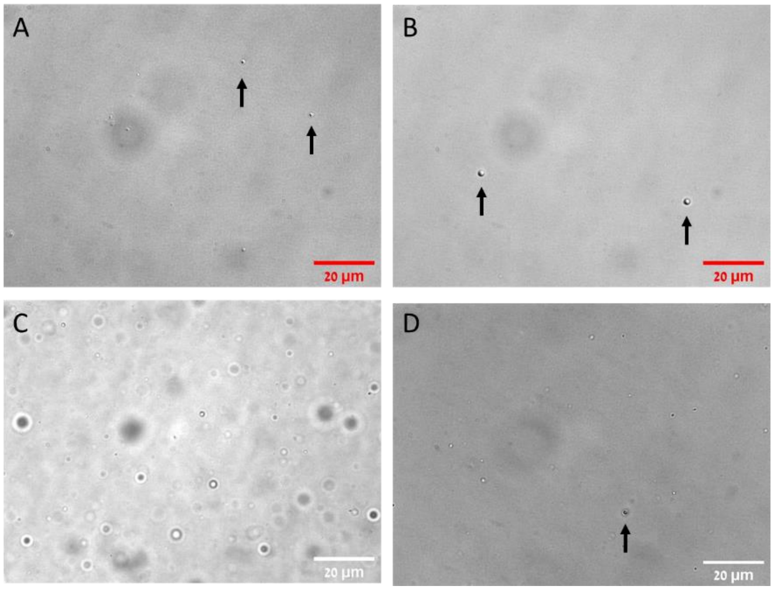

Figure S14. Microscope images of 150 μM PA-12 in A) 0.35 and B) 0.5 mg/ml polyU. C) and D) 50 and 30 μM PA-12 in 1 mg/mL polyU respectively. The arrows show liquid droplets.

9. CW-EPR simulation of PA-12/RNA mixtures

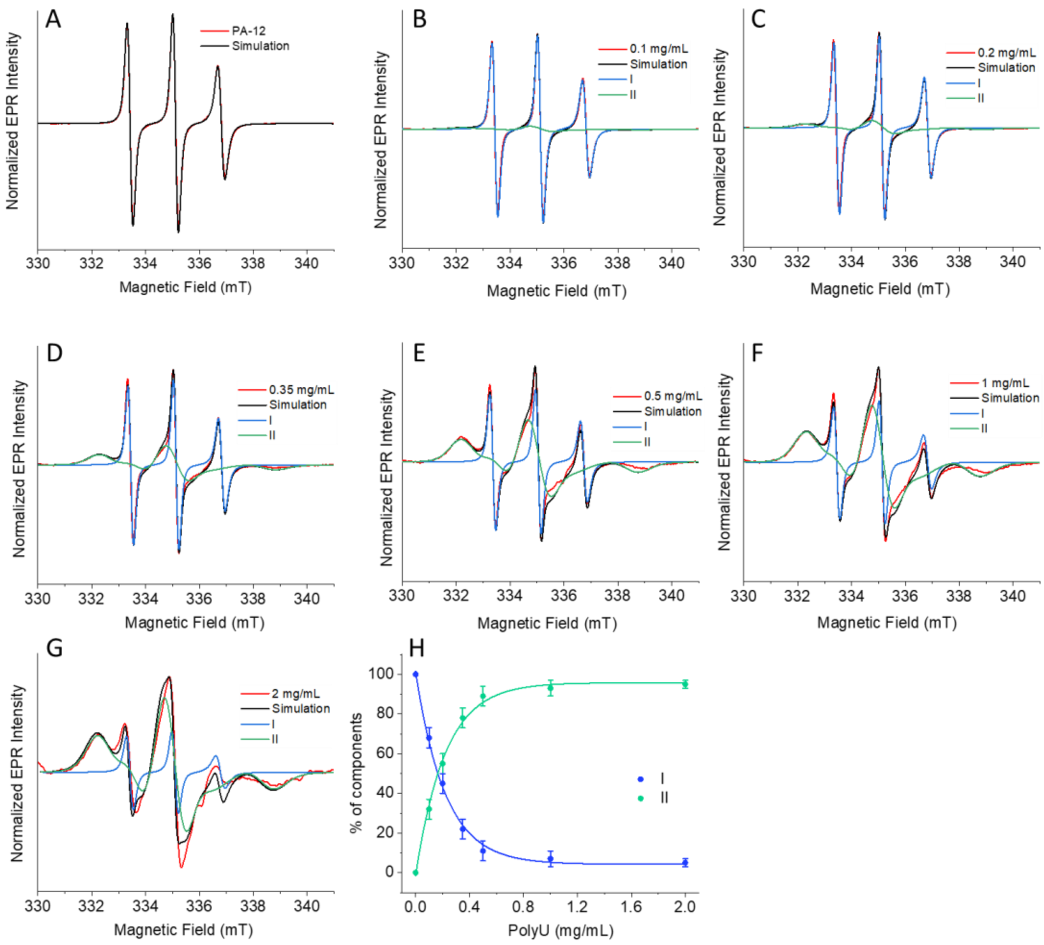

Figure S15. A)-G), Simulation of the CW-EPR spectra of PA-12 in the presence of different polyU concentrations obtained with the 'Chili' routine of Easyspin.[2] Experimental spectra are in red, simulated spectra are in black and individual fast (I) and slow (II) motion components are in blue and green, respectively. For A)-F) the concentration of PA-12 is 150 µM and in G) 100 µM. H) Relative amounts of components I and II as obtained from the simulation of EPR spectra at as a function of polyU concentration.

Table S1. Rotational correlation time (τ_c) and relative population of slow (**II**) and fast motion (I) component from the simulation of CW EPR spectra with different polyU concentration.

PolyU concentration (mg/mL)/PA concentration (μM)	τ_c and % of components	
	**I**	**II**
0/150	0.48 ± 0.1 ns (100%)	0%
0.1/150	0.46 ± 0.1 ns (68 ± 5 %)	20.4 ± 4.6 ns (32 ± 5 %)
0.2/150	0.38 ± 0.1 ns (45 ± 5 %)	20 ± 4.5 ns (55 ± 5 %)
0.35/150	0.39 ± 0.1 ns (22 ± 5 %)	19.1 ± 3.2 ns (78 ± 5 %)
0.5/150	0.43 ± 0.1 ns (11 ± 5 %)	18.2 ± 4.2 ns (89 ± 5 %)
1/150	0.46 ± 0.1 ns (7 ± 4%)	15.6 ± 4.4 ns (93 ± 4%)
2/100	0.57 ± 0.12 ns (5 ± 2%)	13.5 ± 2.3 ns (95 ± 2 %)

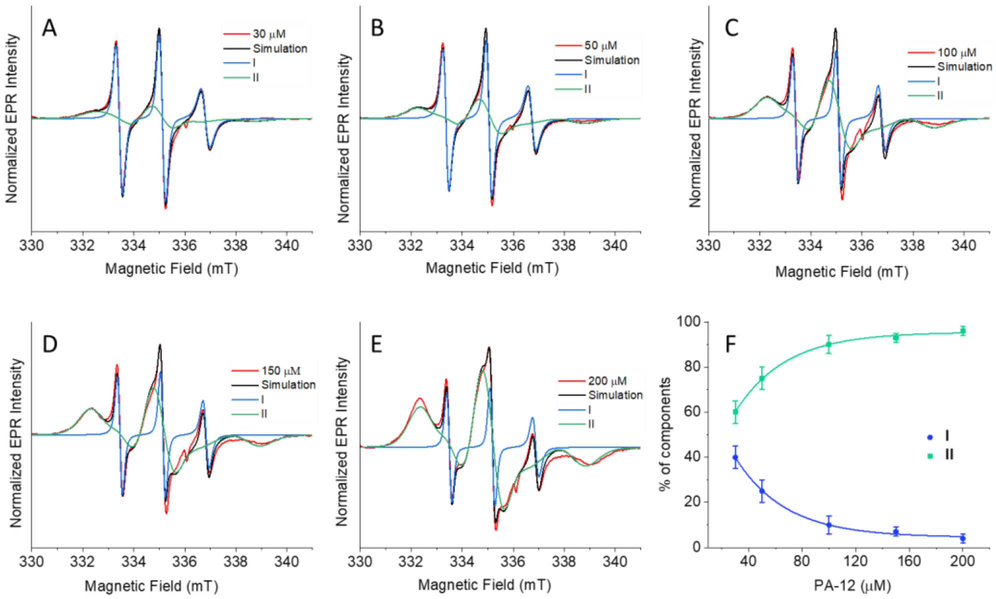

Figure S16. A)-E) Simulation of the CW-EPR spectra at different concentration of PA-12 and fixed concentration of polyU (1 mg/mL). F) relative amounts of components I and II as a function of PA-12 concentration as obtained from the simulation of EPR spectra.

Table S2: Rotational correlation time (τ_c) and relative population of slow (II) and fast motion (**I**) component from the simulation of CW EPR spectra with different PA-12 concentration at 1 mg/mLpolyU concentration.

PA concentration (µM)	τ_c and % of components	
	**I**	**II**
30	0.75 ± 0.05 ns (40 ± 5 %)	9.6 ± 1.4 ns (60 ± 5 %)
50	0.69 ± 0.13 ns (25 ± 5 %)	10.6 ± 1.9 ns (75 ± 5 %)
100	0.53 ± 0.07 ns (10 ± 4 %)	11.6 ± 1.6 ns (90 ± 4 %)
150	0.52 ± 0.12 ns (7 ± 2 %)	11.9 ± 1.4 ns (93 ± 2 %)
200	0.52 ± 0.12 ns (4 ± 2 %)	13.5 ± 2.3 ns (96 ± 2 %)

10. Echo intensity and DEER data of PA-12 with and without polyU

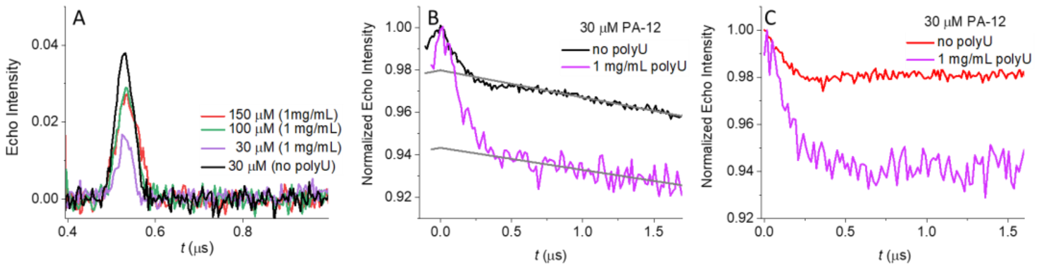

Figure S17. Echo intensity of 30, 100 and 150 µM PA-12 in 1 mg/mL polyU compared with 30 µM PA-12 in the absence of polyU. B) DEER trace of 30 µM PA-12 in the presence of 1mg/mL polyU and in the absence of polyU. Background decay curves are in grey. C) Background subtracted data from B).

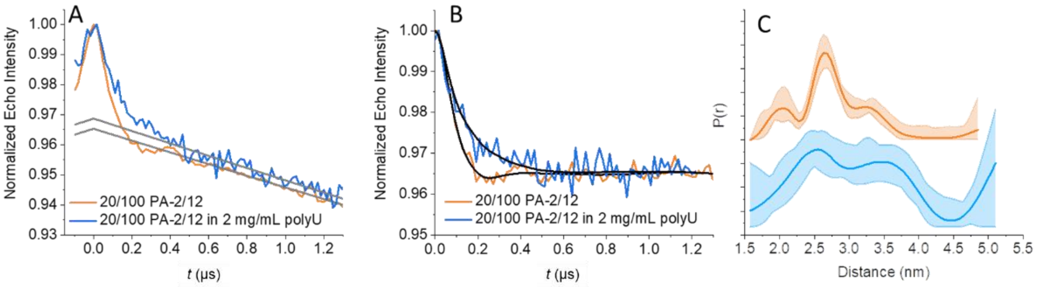

Figure S18. A) Primary and DEER trace of spin diluted PA-2/12 in 2 mg/mL polyU and in the absence of polyU. Labeled and total PA concentrations were 20 and 100 μM respectively. Background decays are shown in grey. B) Background corrected DEER traces with fits (in black) C) The corresponding distance distribution from the fit.

---

[1] Yang Y, Chen SN, Yang F, Li XY, Feintuch A, Su XC, Goldfarb D. In-cell destabilization of a homodimeric protein complex detected by DEER spectroscopy. *Proc Natl Acad Sci U S A*. **2020**,*117*(34),20566-20575.

[2] Stoll, S.; Schweiger, A., EasySpin, a comprehensive software package for spectral simulation and analysis in EPR. *J. Magn. Reson.* **2006,** *178* (1), 42-55.
